# Changes in hepatitis B virus antibody titers over time among children: a single center study from 2012 to 2015 in an urban of South Korea

**DOI:** 10.1186/s12887-017-0924-7

**Published:** 2017-07-14

**Authors:** Kyeong Hun Lee, Kyu Seok Shim, In Seok Lim, Soo Ahn Chae, Sin Weon Yun, Na Mi Lee, Young Bae Choi, Dae Yong Yi

**Affiliations:** 1 Department of Pediatrics, Chung-Ang University Hospital, 102 Heukseok-ro, Dongjak-gu, Seoul, 06973 Republic of Korea; 2 College of Medicine, Chung-Ang University, Seoul, Korea

**Keywords:** Hepatitis B virus, Vaccination, Hepatitis B surface antibody, Children

## Abstract

**Background:**

Hepatitis B virus (HBV) infection is the most common cause of liver disease in endemic areas such as South Korea. After HBV vaccination, hepatitis B surface antibody (HBsAb) titers gradually decrease. Trends in HBsAb titers have not been evaluated among children in South Korea over the past decade.

**Methods:**

We screened 6155 patients (aged 7 months to 17 years) who underwent HBV antigen/antibody testing at Chung-Ang University Hospital from May 2012 to April 2015. Titer criteria were defined as follows: positive, titer ≥100 IU/L; weakly positive, titer 10–99 IU/L; and negative, titer <10 IU/L. We also compared titers before and 1 month after a single booster vaccination.

**Results:**

Of the 5655 patients included, 3016 were male and 5 (0.09%) tested positive for HBV surface antigen. A marked reduction in antibody titer was observed until 4 years of age. Thereafter, the titers showed fluctuating decreases. HBsAb titers reached their lowest levels by 14 years of age. After 7 years of age, 50% of patients tested negative for HBsAb. Simple linear analysis showed that the titer reached levels of <10 IU/L and zero at 12.9 and 13.4 years of age, respectively. 1 month after a single booster vaccination was administered to those who were HBsAb-negative (*n* = 72), 69 children (96%) had developed antibodies while 3 (4%) remained HBsAb-negative.

**Conclusions:**

In conclusion, the continuous reduction in HBsAb titers over time and in each age group was confirmed. The titer level was shown significant decline until age 4. More than half of the sample had negative titers after age 7 years. After booster vaccination, most of child significantly increase titer level.

## Background

Hepatitis B virus (HBV) infection is the most common cause of liver disease in intermediate endemic areas such as South Korea. HBV infection acquired in childhood advances to chronic hepatitis B that requires treatment [1]. If HBV infection is not treated adequately, it can have serious sequelae, such as liver cirrhosis and hepatocellular carcinoma [2–4]. Although both the prevalence of and interest in hepatitis C virus infection have increased recently, HBV infection remains an important issue in terms of viral hepatitis [5].

The main routes of HBV transmission to children are vertical mother-to-child transmission during childbirth and horizontal transmission among family members for children younger than 5 years [6, 7]. The risk of vertical transmission increases with higher maternal HBV DNA levels and with vaginal delivery [8]. The management of HBV carriers and vaccination against HBV are important in endemic areas. Hence, HBV vaccination is included in the national vaccination schedule in South Korea [9, 10].

In South Korea, HBV vaccination was introduced for schoolchildren in 1988 and was included in the national immunization schedule in 1991. Since 1995, routine HBV vaccination has followed the schedule of a dose at 0, 1, and 6 months after birth [6]. In the 1980s, hepatitis B surface antigen (HBsAg) positivity was detected in 6.6–8.6% of the population. After the nationwide vaccination program was introduced in 1995, HBV prevalence decreased dramatically, especially among children younger than 10 years old (prevalence = 0.2%) [11]. The prevalence of HBsAg positivity also decreased among women aged 20–39 years, from 5.48% in 1998 to 2.34% in 2010 for those in their 20s, and from 6.15% in 1998 to 3.85% in 2010 for those in their 30s [12]. Although HBV carriage has decreased substantially, especially among women of childbearing age, it remains the main cause of liver disease, and bears significant social and medical costs. Therefore, it is important to confirm recent trends in hepatitis B surface antibody (HBsAb) positivity and to determine the effects of booster vaccination.

Studies related to the generation of the HBV vaccine and its antibodies have been published, with some reports from Korea [13, 14]. However, no large-scale studies on this issue have been published in the last 10 years. Therefore, in this study, we determined the most recent HBsAg and HBsAb prevalence rates, and investigated the changes in antibody titers according to age in a single urban area in South Korea.

## Methods

We conducted a retrospective, observational, hospital-based study using medical records. A total of 6155 hospitalized pediatric patients, aged between 7 months and 17 years, who underwent HBsAg/antibody (Ab) testing at Chung-Ang University Hospital from March 2012 to April 2015 were eligible for inclusion. Exclusion criteria were as follows: children who had a confirmed record of having received a booster HBV vaccination, children with a birth weight < 2 kg whose first HBV vaccination was delayed by more than 1 month, children with underlying diseases such as liver disease or immune disorders, and children in whom HBsAg/Ab titers were confirmed using only a qualitative test. As administration of HBIG does not inhibit the production of HBsAb, it was excluded from the standard except whether HBIG was administered or not [15]. Consequently, 5655 children were included in this study. Children were categorized into 18 12-month age groups (the only exception was the group of “0-year-olds,” whose age ranged from 7 to 11 months).

A vaccine (0.5 ml) using purified hepatitis B surface antigen protein (EUVAX B INJⓢ 10 mcg/0.5 ml) was injected intramuscularly. Basic inoculation was conducted at 0, 1, and 6 months after birth as a national project. In case of maternal hepatitis B carrier, vaccination and HBIG were administered immediately after birth. Under 10 years old, A booster vaccination schedule was administered at the same dose if necessary, following testing for HBsAg/Ab positivity. Over 10 years old, booster vaccination used doubled dose [6]. An additional test for HBsAg/Ab was performed a month after booster vaccination. All data including booster vaccination were retrospectively confirmed.

The sample was processed based on the hospital protocol. In our hospital, HBsAg/Ab test is carried out at the time of initial blood collection in all hospitalized patients. Blood specimens were refrigerated at 4 °C, and tests were performed within 2 days of sample collection. After centrifugation, serum samples were used for HBsAg/Ab testing. For enzyme immunoassays, we used the chemiluminescent immunoassay method. The samples were analyzed using the ARCHITECT *i*2000SR immunoassay analyzer (Abbott Diagnostics). Titer criteria were defined as follows: positive, titer ≥100 IU/L; weakly positive, titer 10–99 IU/L; and negative, titer <10 IU/L [16, 17]. Booster vaccines were administered to children with confirmed negative results. HBsAb titers were measured 1 month after receiving a single booster vaccination in this subgroup of children, to assess immunologic memory.

All data processing and analysis were conducted using PASW Statistics software, version 18.0 (SPSS Inc., Chicago, IL, USA). We conducted frequency analyses and ANOVA to investigate relationships between HBsAb and other factors (age, gender, AST, ALT), and a simple linear regression with curve estimation to investigate changes in HBsAb according to age. Chi-square tests and t-test were used to compare the data between groups. A *p* value <0.05 was considered statistically significant.

This study was approved by the Institutional Review Board of Chung-Ang University Hospital (C2015128).

## Results

### Prevalence of HBV infection

Data from 5655 children were analyzed. Five children (3 boys and 2 girls; 0.09%) had positive HBsAg test results. All 5 children had negative HBsAb titers and each of their mothers was a confirmed HBV carrier. HBV infection had previously been confirmed in 2 of the 5 children (they were being actively followed-up), but was newly identified in the other 3. Of the 3 newly confirmed cases, one patient was lost to follow-up, one had advanced disease and was attending on-going follow-up evaluations, and one patient, confirmed to be in the replication phase with immune clearance, had been receiving treatment for 1 year. Of these children, 2 were in the 1-year-old group, while the other children were in the 2-year-old, 3-year-old, and 9-year-old groups.

### Characteristics of the patient group

Of the 5650 confirmed HBsAg-negative children (3013 boys and 2637 girls), 1909 (33.8%) had positive HBsAb titers, 2262 (40.0%) had weakly positive titers, and 1479 (26.2%) had negative titers (Table 1). Among the boys, 1063 (35.3%) had positive HBsAb titers, 1189 (39.5%) had weakly positive titers, and 761 (25.2%) had negative titers. The corresponding figures among the girls were 846 (32.1%), 1073 (40.7%), and 718 (27.2%), respectively.

**Table 1:** Characteristics of the children according to hepatitis B antibody titer

	Total	Positive (>100 IU/L)	Weakly positive (10–100 IU/L)	Negative (<10 IU/L)	*p* value
Age (months)	48.21 ± 46.22	25.12 ± 28.74	46.45 ± 40.28	80.71 ± 53.54	<0.001*
Patients	5650	1909 (33.8%)	2262 (40.0%)	1479 (26.2%)	-
Male	3013	1063 (35.3%)	1189 (39.5%)	761 (25.2%)	0.032*
Female	2637	846 (32.1%)	1073 (40.7%)	718 (27.2%)	
AST (IU/L)	43.13 ± 56.08	47.84 ± 56.80	43.17 ± 62.67	36.99 ± 42.18	<0.001*
ALT (IU/L)	23.95 ± 62.28	25.75 ± 49.07	24.15 ± 71.21	21.31 ± 62.85	0.117

Data are expressed as mean ± standard deviation or n (%)

*ALT* alanine aminotransferase, *AST* aspartate aminotransferase

*p value was statistically significant at <0.05

The overall mean age of participants was 48.2 months. The mean ages of children with positive, weakly positive, and negative HBsAb titers were 25.1 months, 46.4 months, and 80.71 months, respectively. Older age was significantly associated with having a negative HBsAb titer (*p* < 0.001). A cross-tabulation analysis demonstrated a slight male preponderance in the antibody-positive group and a slight female preponderance in the antibody-negative group; this difference was statistically significant (*p* = 0.032). The differences between the groups in terms of mean age and aspartate aminotransferase (AST) level were also statistically significant (Table 1). However, in the case of AST, the higher the age, the lower the average tended to be, but the result was derived. When we calculated the average of AST/ALT for those <5 years old and those ≥5 years old, only AST was significantly higher in those <5 years of age (AST: 46.42 versus 33.88, *p =* 0.00) (ALT: 24.40 versus 22.68, *p* = 0.36). Since positive group is younger, AST seems to have come out more meaningfully higher.

### Frequency analysis – changes in HBsAb titer with age

**Fig. 1:**
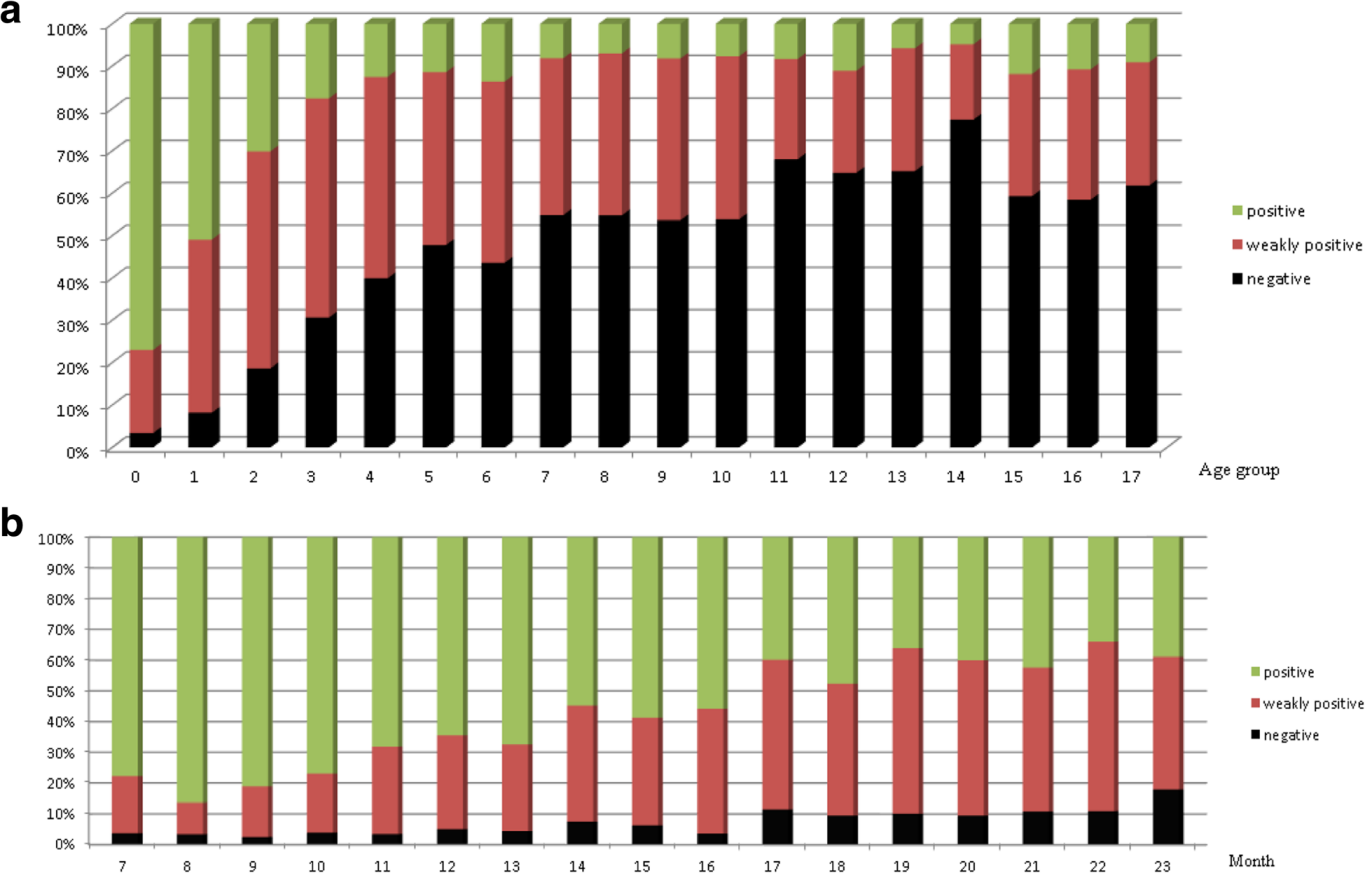
Distribution of hepatitis B surface antibody titer group by age group (**a**) The positive rate was highest in the 0-year-old and 1-year-old age groups and the weakly positive rate was highest in the 2-year-old to 4-year-old age groups; the negative rate was highest thereafter. (**b**) In the most recent 24 months, antibody titer was categorized by month. Positive ratios were observed in at least 50% of children up to 16 months, and in less than 50% thereafter

There was a statistically significant difference in the average HBsAb titer between boys and girls (*p* = 0.002), with boys having higher titers than girls. However, this difference was no longer observed when further stratified by age (Table 2). The positive rate was highest in children <2 years old, the weakly positive rate was greatest in children aged 2–4 years old, and the negative rate was highest in children >5 years. Positive ratios were observed in at least 50% of children up to 16 months of age. Thereafter, <50% of children demonstrated HBsAb titer positivity (Fig. 1).

**Table 2:** Comparison of the hepatitis B antibody titer according to age and sex

Age group	Number of patients	Antibody titer (IU/L)			*p* value
		Overall	Boys	Girls	
0 (7 m–1 yr)	735	456.14 ± 376.04	476.19 ± 382.34	432.65 ± 367.05	0.119
1 (1–2 yr)	1522	241.91 ± 299.15	253.49 ± 305.12	226.70 ± 290.65	0.083
2 (2–3 yr)	890	116.71 ± 185.38	108.24 ± 177.79	125.46 ± 192.71	0.166
3 (3–4 yr)	558	75.98 ± 156.05	81.71 ± 169.15	68.85 ± 138.09	0.334
4 (4–5 yr)	463	50.89 ± 107.57	52.02 ± 107.19	49.43 ± 108.32	0.797
5 (5–6 yr)	297	52.37 ± 137.19	51.59 ± 129.58	53.18 ± 145.09	0.921
6 (6–7 yr)	243	65.19 ± 156.03	71.49 ± 170.57	57.82 ± 137.49	0.497
7 (7–8 yr)	135	33.34 ± 97.52	36.29 ± 125.40	29.98 ± 50.26	0.709
8 (8–9 yr)	157	25.25 ± 44.78	30.46 ± 55.98	22.27 ± 36.91	0.272
9 (9–10 yr)	110	33.37 ± 71.71	25.24 ± 65.27	39.66 ± 76.25	0.298
10 (10–11 yr)	104	39.62 ± 119.40	18.12 ± 34.20	54.77 ± 151.95	0.124
11 (11–12 yr)	72	49.89 ± 169.40	58.61 ± 174.79	41.18 ± 165.85	0.666
12 (12–13 yr)	54	51.03 ± 148.81	38.62 ± 70.26	61.72 ± 193.47	0.574
13 (13–14 yr)	69	29.89 ± 101.35	21.72 ± 44.60	38.80 ± 139.63	0.488
14 (14–15 yr)	62	17.73 ± 49.45	27.23 ± 65.25	11.74 ± 35.90	0.232
15 (15–16 yr)	59	62.31 ± 185.49	38.99 ± 66.50	84.84 ± 251.94	0.347
16 (16–17 yr)	65	44.32 ± 133.60	45.38 ± 160.66	42.72 ± 80.34	0.938
17 (17–18 yr)	55	46.40 ± 148.88	43.00 ± 93.24	50.49 ± 198.32	0.855
Total	5650		176.66 ± 281.93	154.45 ± 260.08	0.002

Data are expressed as the mean ± standard deviation

**Fig. 2:**
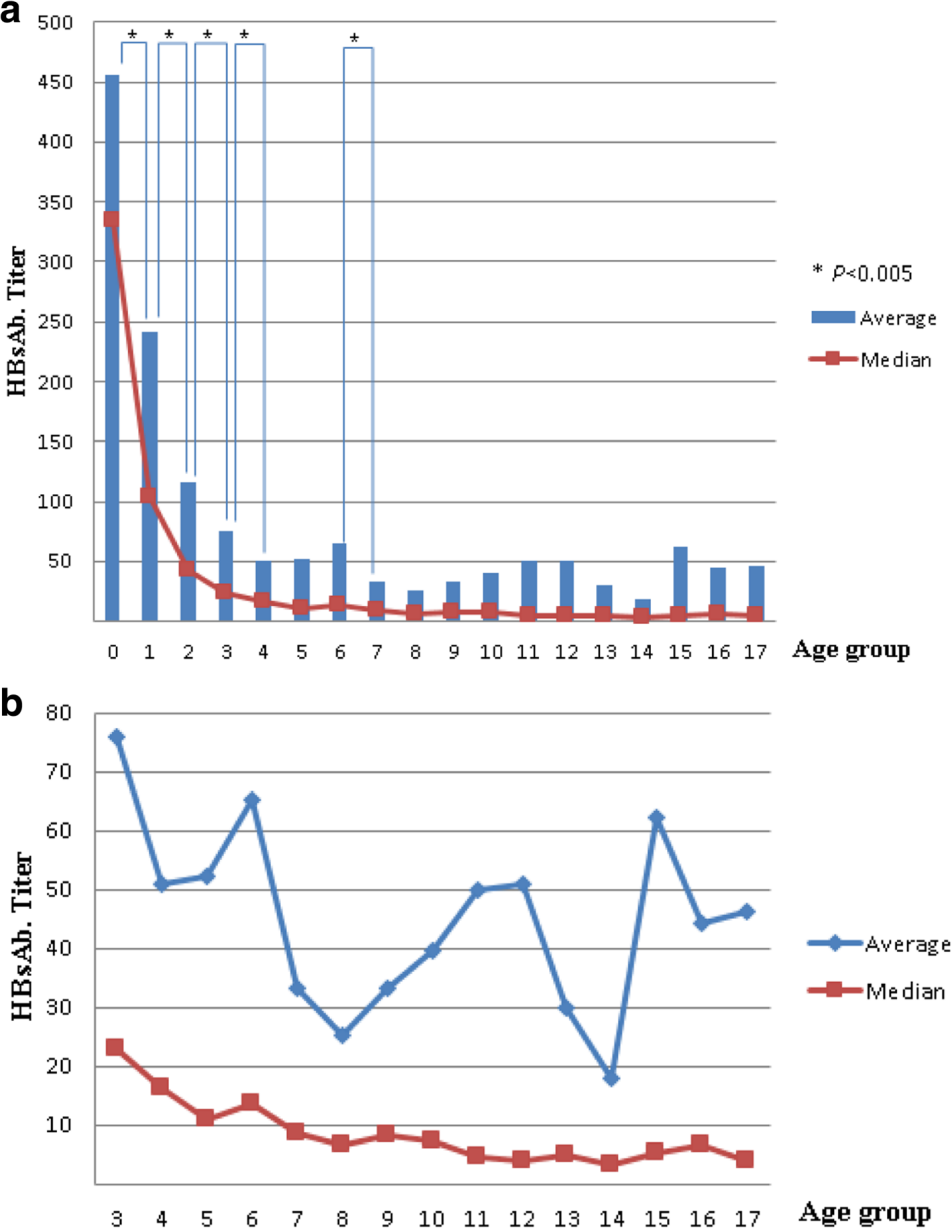
Change in the average and median value of hepatitis B surface antibody titer by age group (**a**) The average titer declined significantly until the 4-year-old age group, and between the 6-year-old and 7-year-old age group. The antibody average titer was lowest in the 14-year-old age group, followed by the 8-year-old age group. After 14 years of age, the titer appears to rise. (**b**) The graph shows values extracted separately in groups older than the 3-year-old group. The median titer value is less than 10 after the 7-year-old group

The average titer continued to decline until the group of 4-year-olds. Thereafter, the fluctuations were not statistically significant. However, a statistically significant reduction was observed between the 6-year-old and 7-year-old age groups. The average HBsAb titer was lowest in the 14-year-old group, followed by the 8-year-old group. The titers began to rise after the age of 15 years. The median titer value dropped to <10 IU/L in the 7-year-old group. Hence, >50% of children had negative HBsAb titers after 7 years of age. The median titer value was <100 IU/L (indicating seroconversion from positive to weakly positive) at 17 months of age (Fig. 2).

### Simple linear regression – changes in HBsAb titers with age

**Fig. 3:**
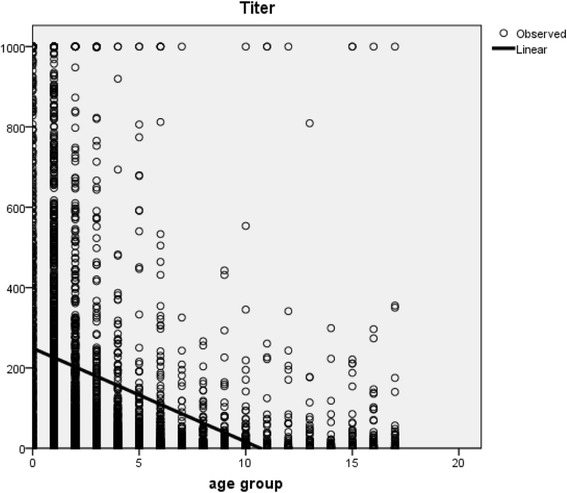
Simple linear regression–changes in hepatitis B surface antibody titer with age

Linear regression equations were estimated for the correlation between age and HBsAb titer, with a slope of −18.969 and a constant of 255.082 (y = −18.969푥 + 244.082). The initial titer was 224 IU/L, reaching a level of 100 IU/L at 7.9 years old, a level of 10 IU/L at 12.9 years old, and a level of zero at 13.4 years old (Fig. 3).

### Evaluation of the booster effect

Seventy-two HBsAb-negative children (only 4.9% of children were confirmed as HBsAb-negative) received a single booster vaccination. Titer tests were performed a month after receiving the booster vaccination. At this time, 46 (64%) were positive, 23 (32%) were weakly positive, and 3 (4%) were negative. Only 3 children received a booster vaccination, according to the schedule of 0, 1, and 6 months. All 3 showed positive findings a month after completing the booster schedule.

## Discussion

The HBsAg positivity rate, reported as 0.12% among teenagers in 2010 [12], was 0.09% in our population of hospitalized chidren. This decrease in the prevalence of HBV infection in children is due to the reduction in prevalence of maternal HBV infection. Moreover, pregnant women are screened for HBV infection, and HBV immunoglobulin is administered immediately after birth to infants born to mothers who are HBV carriers. This reduces the possibility of vertical transmission. In addition, in South Korea, routine infant vaccination is now provided [12].

Compared with other countries, the observed prevalence was lower in the present study than in Papua New Guinea (2.3% in 2012–2013) and Tajikistan (0.4% in 2010), which are high endemic areas [18, 19]. In Henan Province in China, the HBV prevalence among children in 2012 was 0.8% [20], demonstrating that HBsAg positivity is reduced by the introduction of routine vaccination. Prior to routine vaccination, the prevalence was higher in China than in South Korea. In Japan, the HBsAg positivity rate was 0.17% in the period 2005–2011 [21]. However, Japan does not provide routine vaccination; rather, vaccination is administered selectively to groups at risk.

Except for vaccination status, risk factors for high rates of HBV infection or low immunologic responses include: maternal hepatitis B carrier; a family member who is a hepatitis B carrier; previously having blood transfusion history; being a liver transplant recipient; having an underlying malignancy/liver disease (such as non-alcoholic fatty liver disease), rheumatologic disease (such as juvenile idiopathic arthritis), or steroid-resistant nephrotic syndrome; using of steroid therapy; being born preterm or low birth weight; and being a healthcare worker. Children with an autoimmune disease show a higher immunologic response [22–32]. Among adults, the risk factors for a low immunologic response are reported to be age (≥40 years), male sex, body mass index ≥25 kg/m^2^, being a smoker, and having concomitant disease [33].

In a study in Korea conducted 10 years ago, it was reported that the levels of antibodies gradually decreased until the age of 12 years, after which point they rose [13]. In the present study, antibody titers post-vaccination showed a steady decline up to 4 years of age. Thereafter, the changes were more fluid. From the age of 7 years, >50% of children had a negative titer. The linear regression analysis showed a decrease in antibody titer with increased age, reaching a titer <10 IU/L at 12.9 years and a titer of 0 at 13.4 years. Therefore, after the age of 12–14 years, children should receive booster HBV vaccination, as it is possible that by this age most children have become antibody negative. However, in the present study, the average titer value was observed to be lowest in the 14-year-old age group, and thereafter, it gradually increased. There are several hypotheses to explain this phenomenon. First, in Seoul, children entering their first year in junior high school (11–12 years) receive a regular medical examination, including assessment of HBsAg/Ab levels. This may increase the chance of receiving a booster inoculation. Second, the vaccination guidelines published by the Health and Welfare Vaccination Deliberation Committee in 1997, based on those of the Academy of Pediatrics, were changed. Guidance to provide inoculation with a booster hepatitis B vaccine only in special cases was eliminated. Therefore, it is possible that children older than 14 years had received a booster vaccine. Even in the 10 years prior to this study, antibody titers were shown to rise after the age of 12 years. The authors explained that this may be due to the overlap of the study period with changes in the national vaccination program’s target age from school age children to infants [13]. In the present study, all participants had been vaccinated according to the infant schedule (at 0, 1, and 6 months of age). Hence, the previous hypothesis is not applicable. In Iran, the antibody-positivity rate was 90% at 1 year following vaccination in the neonatal period, but then decreased over time in accordance with age to 48.9% at 18 years. However, the lowest antibody positivity rate (35.7%) was observed in those aged 11–15 years old. In that study, the possible explanation for this phenomenon was the implementation of a national vaccination project [17]. Countries in which there are not national vaccination programs showed a sustained reduction in HBsAb titer with age, such as Saudi Arabia, China, Brazil, Taiwan, Hong Kong, Alaska, and Egypt [34–38].

After HBV vaccination, HBsAb titers decrease over time, but immunologic memory was maintained for at least 10 years [39, 40]. Even though HBsAb titers decrease, infants with normal immunologic function develop a preventive effect against infection. Hence, administering boosters to those who have completed a 3-dose immunization program is not recommended [6]. Nonetheless, non- or low-responders who complete the planned vaccination schedule demonstrate better responses after receiving re-vaccinations [30]. The current vaccination guidelines state that if a vaccinated person tests antibody negative, it is most likely that this is due to reductions in antibody concentrations over time, rather than being an indication of vaccine non-response. Therefore, instead of administering 3 doses for re-vaccination to achieve a sharp rise in antibody titer, it is recommended that, due to immune memory, only a single dose should be administered [6]. In the present study, because booster vaccination is not essential, the 4.9% of the children who were titer negative received booster inoculation. Children who were HBsAb-negative received a single booster dose of HBV vaccine, and immune response was confirmed after a month. This supports the hypothesis that a decrease in HBsAb concentration, rather than vaccine non-response, led to the observed negative antibody status.

The present study had a large sample size (>5000 people), and reveals the most recent trends in hepatitis B antibody titers of children in South Korea. However, our study was limited by not being representative of children in South Korea as a whole, because the work was conducted among urban children living in Seoul and all of the included children were admitted to a hospital.

## Conclusion

Our study shows that HBsAb titers in children decrease over time, with 50% of children >7 years old being seronegative and HBsAb titers reaching zero by 13 years. Hence, if a child older than 7 years requires blood tests, while either in the hospital or as part of regular medical examination, it would be worth considering testing for HBV markers. In particular, routine HBsAb testing should be considered for children aged 12–14 years who did not receive a booster HBV immunization. Lastly, once HBsAb-negative children have received additional HBV vaccination, an attempt should be made to confirm reactivity, as this will be cost-effective.

Future studies of large cohorts are required to determine whether type-specific scheduled vaccination or cross-inoculation scheduled vaccination would increase the effectiveness of vaccination.

## Abbreviations

ALT: Alanine aminotransferase
AST: Aspartate aminotransferase
HBsAb: Hepatitis B surface antibody
HBsAg: Hepatitis B surface antigen
HBV: Hepatitis B virus
